# Optimization and kinetic study of methyl laurate synthesis using ionic liquid [Hnmp]HSO_4_ as a catalyst

**DOI:** 10.1098/rsos.180672

**Published:** 2018-09-12

**Authors:** Benyong Han, Wudi Zhang, Fang Yin, Shiqing Liu, Xingling Zhao, Jing Liu, Changmei Wang, Hong Yang

**Affiliations:** 1 Faculty of Energy and Environmental Science, Yunnan Normal University, No. 768, Juxian Street, Chenggong District, Kunming 650500, Yunnan, People's Republic of China; 2 Faculty of Life Science and Technology, Kunming University of Science and Technology, 727 South Jingming Road, Chenggong District, Kunming 650500, Yunnan, People's Republic of China

**Keywords:** response surface, ionic liquid [Hnmp]HSO_4_, esterification, methyl laurate, kinetic study

## Abstract

Methyl laurate was synthesized from lauric acid (LA) and methanol via an esterification reaction using ionic liquids (ILs) as catalysts. The efficiencies of three different catalysts, 1-methylimidazole hydrogen sulfate ([Hmim]HSO_4_), 1-methyl-2-pyrrolidonium hydrogen sulfate ([Hnmp]HSO_4_) and H_2_SO_4_, were compared. The effect of the methanol/LA molar ratio, reaction temperature, reaction time and catalyst dosage on the esterification rate of LA was investigated by single-factor experiments. Based on the single-factor experiments, the esterification of LA and methanol was optimized using response surface methodology. The results showed that the most effective catalyst was the IL [Hnmp]HSO_4_. The optimal conditions were as follows: [Hnmp]HSO_4_ dosage of 5.23%, methanol/LA molar ratio of 7.68 : 1, reaction time of 2.27 h and reaction temperature of 70°C. Under these conditions, the LA conversion of the esterification reached 98.58%. A kinetic study indicated that the esterification was a second-order reaction with an activation energy and a frequency factor of 68.45 kJ mol^−1^ and 1.9189 × 10^9^ min^−1^, respectively. The catalytic activity of [Hnmp]HSO_4_ remained high after five cycles.

## 1. Introduction

Esters obtained from esterification reactions have attracted widespread attention due to their extensive applications in the food, cosmetic, plasticizer, pharmaceutical, plastic and chemical industries [1,2]. The most significant and valuable product obtained by the esterification of long-chain fatty acids is biodiesel [3,4], which has many advantages, i.e. biodegradability, cleaner engine emissions, low viscosity, renewability, good miscibility with fossil diesel and superior lubricating properties, which make it an outstanding substitute for or additive to conventional diesel fuels [5,6]. The most common way to produce biodiesel is from vegetable oil/animal fat by transesterification of triglycerides with a short-chain alcohol (methanol/ethanol) in the presence of alkali or acid catalysts. In the light of the oil feedstock, biodiesel is reported to be costly, and its price is two times higher than that of petroleum diesel [7]. Therefore, in an attempt to improve the economic feasibility of biodiesel, raw materials of non-edible oils such as waste cooking oil and by-products of refining vegetable oils have been considered because they are available at a reasonable price [8,9], which is an effective pattern to reduce the production cost. However, there is a high concentration of free fatty acids (FFAs) in non-edible oils. To achieve a reasonable conversion to biodiesel, the FFAs of non-edible oils should be converted to fatty acid methyl esters by esterification with methanol. In general, the esterification reaction can be catalysed by a homogeneous acid [10], such as H_2_SO_4_, HCl and organic sulfonic acids, which are traditionally selected as the acid catalyst [7]. However, homogeneous acid-catalysed reactions can suffer from environmental and corrosion problems, which together negatively impact their applications in continuous processing, especially during neutralization and separation steps. Other issues, such as unfavourable by-products and difficult catalyst recovery and reuse [11], also hinder the large-scale production of biodiesel from high FFA oils using homogeneous acid catalysts. Thus, researchers have attempted to develop effective and eco-friendly catalysts for the esterification of FFAs to provide biodiesel. For example, solid super acids [12], heteropolyacids [13], metal oxides [14], zeolites [15], molecular sieves [16] and enzymes [17] have been used for biodiesel production.

Ionic liquids (ILs) have been applied as green solvents and catalysts because of their good thermal stability, outstanding solubility, negligible volatility, tuneable physical and chemical properties, and reusability [18,19]. Esterification reactions catalysed by ILs are attracting increasing attention because they enable easy product separation, process equipment downsizing, reduced waste water generation, and, consequently, decreased environmental influence and process costs. Accordingly, the use of IL catalysts in esterification reactions is exceedingly important for developing cleaner and more economical processes for biodiesel production. For example, Xian and co-workers reported good catalytic activity for the Brønsted acidic IL [NMP][CH_3_SO_3_] in the esterification of various fatty acids and alcohols [20]. Rad-Moghadam and co-workers used efficient NMP-ClSO_3_H salt ILs in the synthesis of δ-sultones [21]. Huang *et al.* [22] reported a dehydration of fructose into 5-hydroxymethylfurfural promoted by ILs [Bmim]Cl and [NMP][CH_3_SO_3_] in ethanol. In our previous study [23], the catalytic activity of the acidic IL 1-methyl-2-pyrrolidonium hydrogen sulfate salt ([Hnmp]HSO_4_) in the synthesis of methyl laurate was studied without removing water during the esterification. Biodiesel synthesis via esterification reactions has been conducted using ILs as catalysts; however, reports of the kinetics of the esterification of lauric acid (LA) are very limited in the literature. Kinetic studies are focused on the influences of temperature on reaction rates, which also critically depend on the type of fatty acid used, the nature and amount of the catalyst used and the temperature.

The kinetics of LA esterification catalysed by IL [Hnmp]HSO_4_ have not been reported previously in the literature. This study is essential for conducting the parametric optimization of the esterification process of LA using response surface methodology (RSM) [24]. In addition, the present work also calculated the kinetics of the esterification process. Specifically, under the condition of removing water with 3A molecular sieves, the esterification of LA with methanol catalysed by [Hnmp]HSO_4_ was investigated. RSM was employed to optimize the catalyst levels, methanol/LA molar ratio and reaction time. Moreover, a kinetic model was proposed, and the kinetic parameters were determined by fitting the model with the experimental results.

## 2. Material and methods

### 2.1. Materials

LA (analytical reagent grade (AR)) was purchased from Sinopharm Chemical Reagent Co., Ltd (Shanghai, China). 1-Methyl-2-pyrrolidone (AR, 99%), acetonitrile (AR), ethyl acetate (AR), ethyl ether (AR) and methanol (AR) were obtained from Aladdin Reagent Co., Ltd (Shanghai, China). Concentrated sulfuric acid (AR, 98%), ethanol (95%, industrial grade), KOH (AR), dipotassium phthalate (AR) were obtained from Sichuan Xilong Chemical Industry Co. Ltd (Chengdu, China). Guaranteed reagent-grade IL [Hmim]HSO_4_ was purchased from Shanghai Chengjie Chemical Co., Ltd 3A molecular sieves were purchased from Guangzhou Chemical Reagent Factory. Deionized water was prepared in our laboratory. All reagents were obtained from commercial sources and used without further purification.

### 2.2. Preparation of Brønsted acidic IL [Hnmp]HSO_4_

In the absence of solvent, 1-methyl-2-pyrrolidone was added to a 250 ml round-bottom flask. Then, a stoichiometric amount of concentrated sulfuric acid (98%) was added dropwise at 0°C, and the mixture was stirred for 1 h at 0°C and then stirred for 24 h at room temperature [25]. The Brønsted acidic IL [Hnmp]HSO_4_ was washed repeatedly with ethyl acetate to remove non-ionic residues and dried under vacuum.

### 2.3. Catalytic testing and measurement of the reaction extension

Weighed amounts of LA, methanol and IL were added to a three-necked round-bottom flask equipped with a reflux condenser, a water separator and a magnetic stirring apparatus. The esterification reaction was typically carried out for a certain amount of time at the desired temperature with vigorous stirring. Then, the reaction mixture became biphasic, and the upper phase, which was mainly the desired methyl laurate, could be isolated simply by decantation; the lower phase, the viscous IL, could be reused after the water in the IL was removed. The product was directly measured by KOH–EtOH titration, and the acid value (AV) was calculated.

AVs were analysed according to AOCS Cd 3d-63 and AOCS Ca 5a-40 [26], which were used to evaluate the rate of esterification (RE). The AV (mg KOH g^−1^) of biodiesel was determined as follows:

2.1 $$\text{AV}=\frac{C(V_{1}-V_{2})\times 56.11}{M},$$

where *C* is the concentration of KOH solution (mol l^−1^); *V*_1_ and *V*_2_ are the volumes of KOH solution consumed for the titrating sample and blank test (ml), respectively; *M* is the weight of the sample (g); and 56.11 is the molar mass of potassium hydroxide (g mol^−1^).

RE was defined as follows:

2.2 $$\text{RE}=\left[\frac{X_{0}-X}{X_{0}}\right]\times 100\text{\%}\;,$$

where *X*_0_ and *X* are the initial and equilibrium AVs of biodiesel, respectively. The initial AV (*X*_0_) of LA is 280.2 ± 2.0 mg KOH g^−1^.

### 2.4. Comparison of different catalysts

For a reaction time of 4 h, a methanol/LA molar ratio of 3 : 1, a catalyst dosage of 5% and a reaction temperature of 100°C, the effects of [Hmim]HSO_4_, [Hnmp]HSO_4_ and H_2_SO_4_ on the LA conversion were investigated. Then, the most suitable catalyst was selected by considering the number of sustainable cycles and the environmental impact.

### 2.5. Single-factor experiments

The effect of [Hnmp]HSO_4_ dosage (1%, 2.5%, 5%, 7.5%, 10%, 12.5%, 15%), methanol/LA molar ratio (1 : 1, 3 : 1, 6 : 1, 9 : 1), reaction temperature (55, 70, 85, 100, 115°C) and reaction time (1, 2, 3, 4, 5, 6,7,8 h) on the LA conversion was studied.

### 2.6. Experimental design and statistical analysis

The optimum conditions for the synthesis of methyl laurate using loaded [Hnmp]HSO_4_ IL as a catalyst were determined by means of RSM. A Box–Behnken experimental design [27,28] was chosen to assess the relationship between the yield of biodiesel and the reactant ratio, amount of IL and time. A total of 17 sets of experiments (12 factorial points and five centre points) were performed according to a 3^3^ Box–Behnken experimental design. The three variables were tested at the three levels by plus signs (+1) associated with high levels, zero (0) indicating centre values and minus signs (−1) associated with low levels. The coded values of these factors were obtained according to equation (2.3) as follows:

2.3 $$x_{i}=\frac{X_{i}-X_{0}}{\Delta X_{i}},$$

where *x_i_* is the independent variable coded value, *X_i_* is the independent variable real value, *X*_0_ is the independent variable real value at the centre point and Δ*X_i_* is (variable at high level − variable at low level)/2. The independent variables and their levels and real values are presented in table 1.

**Table 1.** Parameter levels and coded values used in the experimental design.

factors	symbol	range and level		
		−1	0	+1
amount of catalyst (wt%)	*X*_1_	2.50	5.00	7.50
ethanol/acid molar ratio	*X*_2_	3	6	9
reaction time (h)	*X*_3_	1	2	3

The second-order model equation given by RSM was used to predict the optimum value and analyse the interaction between the variables and the yield of biodiesel, which was the response of the experimental design. The quadratic equation model was described according to the following equation:

2.4 $$Y=\beta_{0}+\sum_{i=1}^{k}\beta_{i}x_{i}+\sum_{i=1}^{k}\beta_{ii}x_{i}^{2}+\sum_{i=1}^{k}\beta_{ij}x_{i}x_{ij}+\varepsilon ,$$

where *Y* is the response variable, *X_i_* is the coded level of the independent variables and the terms *β_0_, β_i_, β_ii_* and *β_ij_* are the regression coefficient, the linear terms, the squared terms for variable *i* and the interaction terms between variables *i* and *j*, respectively. *X_i_, X_ii_* and *X_ij_* represent the linear, quadratic and interactive terms of the coded independent variables, respectively. *k* is the total number of variables and is optimized in the present experiment. *ε* is a random error. The polynomial equation visualized the relationship between the response and experimental levels of each factor and determined the optimum conditions by response surface and contour plots. The coefficient of determination (*R*^2^) could be used to evaluate the accuracy and general ability of the second-order multiple regression models. The significance of the regression coefficient was checked with the value of F-test.

Design-Expert software (V. 8.0.6, Stat-Ease. Inc., USA) was used to analyse the data, perform analysis of variance (ANOVA) and estimate the regression equation.

### 2.7. Kinetics of the esterification process

Acid esterification involves a reversible reaction between a fatty acid and a primary alcohol in the presence of an acid catalyst [29]:

$$\text{RCOOH}+\text{C}{\text{H}}_{3}\text{OH}\Leftrightarrow \text{RCOO}{\text{H}}_{3}+{\text{H}}_{2}\text{O}.$$

The general reaction rate equation is represented as follows:

2.5 $$-\frac{\text{d}C_{A}}{\text{d}t}=k_{1}C_{A}^{\alpha }C_{B}^{\beta }-k_{2}C_{R}^{r}C_{S}^{s},$$

where *C_A_, C_B_, C_R_* and *C_S_* represent the concentrations of LA, methanol, methyl laurate and water, respectively. *k*_1_ and *k*_2_ are the forward and reverse reaction rate constants, respectively. *α, β, r* and *s* are the reaction orders with respect to *A*, *B*, *R* and *S*, respectively [29].

In this work, excess methanol is used to drive the reaction towards the product side. When the methanol to LA molar ratio is sufficiently high, the concentration of methanol is considered constant. Thus, this esterification can be approximately described by as an irreversible reaction. Based on the abovementioned assumptions, the IL catalysed esterification could be simplified by a pseudo-homogeneous equation with *α* as the reaction order as follows:

2.6 $$-r_{A}=-\frac{\text{d}C_{A}}{\text{d}t}=kC_{A}^{\alpha }.$$

The conversion of LA is assumed to be *X* at *t* = 0; hence, the concentration of the reactants can be expressed as *C_A_* = *C_A_*_0_(1 *−* *X*), where *C_A_*_0_ is the initial concentration of LA. Equation (2.6) can be expressed as follows:

2.7 $$-r_{A}=-\frac{\text{d}C_{A}}{\text{d}t}=-\frac{\text{d}(C_{A0}(1-X))}{\text{d}t}=C_{A0}\frac{\text{d}X}{\text{d}t}=k{\left(C_{A0}(1-X)\right)}^{\alpha }.$$

Based on the abovementioned hypothesis, this esterification is a second-order reaction, so we can establish the reaction order *α* = 2. Equation (2.7) can be expressed using an integral transformation as follows:

2.8 $$\frac{1}{(1-X)C_{A0}}=kt,$$

where *k* and *X* are the reaction rate constant and the reaction conversion, respectively. They can be determined by plotting 1/((1 − *X)C_A_*_0_) against *t*. In this kinetic study, the experiments were carried out under the optimal conditions with respect to a reaction time interval of 30 min (30, 60, 90, 120, 150 and 180 min) and four different temperatures (60, 65, 70 and 75°C). All the experiments were repeated three times.

The Arrhenius equation, which expresses the temperature dependence of the reaction rate, was used to determine the activation energy and frequency factor of the esterification process:

2.9 $$k=A{\text{e}}^{-E_{a}/RT},$$

where *A* is the pre-exponential factor, min^−1^; *E* is the activation energy, J mol^−1^; *R* is the gas constant (8.314 J mol^−1^ K^−1^); and *T* is the absolute temperature, K.

By using the integral transformation of equation (2.9), it becomes:

2.10 $$\text{ln}k=\text{ln}A-\frac{E}{R}\left(\frac{1}{T}\right).$$

In a plot of ln*k* versus 1/*T*, the slope and the intercept of the regression line equal *−E*/*R* and ln*A*, respectively. The activation energy and pre-exponential factor are then calculated from those values.

### 2.8. Recycling experiment for [Hnmp]HSO_4_

The IL was recycled after withdrawing the lower layer from the separating funnel. The lower phase was a mixture of the IL, water and unreacted methanol. The water and excess methanol were removed from the mixture by rotary evaporation, and then the IL catalyst [Hnmp]HSO_4_ was further washed with ethyl acetate to remove the organic ester, followed by vacuum drying for 5 h at 80°C. The IL catalyst obtained was used in the next cycle.

## 3. Results and discussion

### 3.1. Effect of three different catalysts on the lauric acid conversion

The efficiency of [Hnmp]HSO_4_ was studied by comparison with other catalysts, such as 1-methylimidazole hydrogen sulfate salt ([Hmim]HSO_4_) and concentrated sulfuric acid (H_2_SO_4_). According to the literature reports [9,25,29,30], imidazolium salts are the most widely investigated ILs and have been applied in many fields [9]. [Hmim]HSO_4_ is a Brønsted acid IL composed of 1-methylimidazole and concentrated sulfuric acid, and H_2_SO_4_ is the most commonly used acid catalyst [29]. IL [Hnmp]HSO_4_ has several advantages: (i) the preparation of [Hnmp]HSO_4_ is very easy, and the cost is low [25,30]. (ii) The IL [Hnmp]HSO_4_ showed good catalytic performance for the esterification of acetic acid with *n*-butanol. The esterification procedure could be carried out at mild temperatures, and the esters produced could be isolated conveniently in high yields and purity [25]. Therefore, the use of [Hnmp]HSO_4_ will reduce production costs. The reaction without catalyst was also carried out to evaluate the conversion gain provided by the catalyst over the non-catalytic reaction performance.

**Figure 1.**
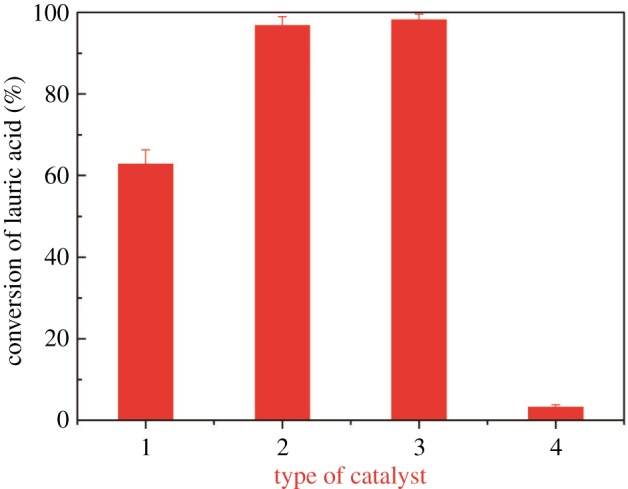
Effects of three different catalysts (1, [Hmim]HSO_4_; 2, [Hnmp]HSO_4_; 3, H_2_SO_4_), as well as without catalyst (4), on the LA conversion (reaction temperature: 100°C, catalyst amount: 5%, molar ratio of methanol to acid: 3 : 1, reaction time: 4 h).

As shown in figure 1, the LA conversion was only 3.25% without using a catalyst. In other words, the chemical reaction takes place very slowly in the absence of a catalyst. Three different catalysts were used separately in the esterification reaction. H_2_SO_4_ had the best catalytic effect, with an LA conversion of 98.23%. The catalytic efficiency of [Hnmp]HSO_4_ was close to that of the conventional catalyst H_2_SO_4_. The worst catalytic efficiency was shown by [Hmim]HSO_4_, for which the LA conversion was only 62.83%. However, H_2_SO_4_ has some disadvantages, such as strong corrosivity, non-recyclability and environmental pollution. The use of [Hnmp]HSO_4_ could overcome these drawbacks.

The performance of various catalysts in the esterification of LA is summarized in table 2, where the response is reported as the LA conversion. Clearly, the IL used in this study exhibited lower catalytic activity in the esterification of LA than some solid catalysts, i.e. ferric alginate [31] and CePW_12_O_40_ [32]. But, the conversion was better than that achieved using montmorillonite K10 [33], MMT-NO_3_ [34] and $\text{S}{\text{O}}_{4}^{2-}/\mathrm{Sn}{\text{O}}_{2}-\text{Si}{\text{O}}_{2}$ [35]. In this study, the IL [Hnmp]HSO_4_ showed some advantages in our case. First, it showed higher conversion rates than the IL [Hmim]HSO_4_. Second, the use of 1-methyl-2-pyrrolidone as a source of cations is more economical than that of 1-methylimidazole and 1-methylpyrrolidine [30]. In addition, the recycling of [Hnmp]HSO_4_ was very easy. Taking all factors into consideration, [Hnmp]HSO_4_ was selected for further research.

**Table 2.** Catalytic performance of different catalysts in the esterification of LA.

catalyst	experimental conditions				conversion (%)
	catalyst dosage (%)	molar ratio	temperature (°C)	time (h)	
ferric alginate	16	16 : 1	65	3	99 [31]
CePW_12_O_40_	7	15 : 1	67	4	97.0 [32]
montmorillonite K10	12	12 : 1	160	2	95.06 [33]
MMT-NO_3_	8	12 : 1	160	2	93.08 [34]
$\text{S}{\text{O}}_{4}^{2-}/\mathrm{Sn}{\text{O}}_{2}-\text{Si}{\text{O}}_{2}$	5	10 : 1	65	4	80.54 [35]
[Hnmp]HSO_4_	5	3 : 1	100	4	96.78

### 3.2. Single-factor experiments

**Figure 2.**
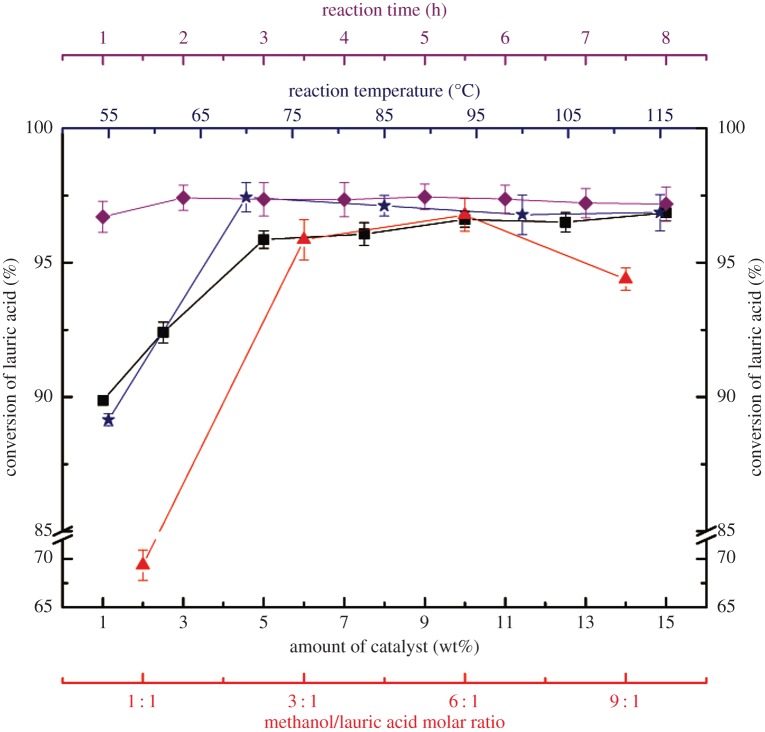
Effects of four factors on LA conversion. (Filled square: amount of catalyst ([Hnmp]HSO_4_), filled triangle: methanol/LA molar ratio, filled star: reaction temperature and filled diamond: reaction time.)

The effect of the [Hnmp]HSO_4_ dosage, methanol/LA molar ratio, reaction temperature and reaction time on the LA conversion was analysed and is shown in figure 2. The effect of different catalyst dosages on LA conversion was studied first. To find the optimum value of the amount of catalyst, the other three variables were fixed. According to the value in the literature [25], the temperature was set to 100°C. To make the reaction go to completion, the reaction time was set to 4 h, and the molar ratio of methanol to acid was set to 3 : 1. Generally, the catalyst dosage should influence the reaction rate and the LA conversion. In this work, a succession of experiments were carried out using different dosages of [Hnmp]HSO_4_. As shown in figure 2, initially, the rate of the esterification reaction sharply increased with increasing [Hnmp]HSO_4_ dosage, reaching 95.85% LA conversion at a 5% dosage; however, there was no significant improvement beyond 5%. Considering the preparation cost, 5% was chosen as the optimum catalyst dosage.

Subsequently, the effect of different molar ratios on LA conversion was studied. The other three variables were also fixed. An excess of the reactant methanol is necessary for the esterification of LA because it can increase the rate of methanolysis. The molar ratio of methanol to LA varied from 1 : 1 to 9 : 1, and the conversions obtained are shown in figure 2. The greater the amount of methanol added, the higher the conversion of LA to methyl laurate obtained in the same reaction time. The LA conversion rapidly increased with molar ratio up to 6 : 1 and decreased thereafter. The highest conversion of LA achieved was 96.87% with a methanol to LA molar ratio of 6 : 1 in 4 h. Further increases in this molar ratio did not result in an increase in the conversion, which could be because of the excess methanol, which diluted the concentration of hydrogen ions in the reaction mixture. This result was in good agreement with the literature reported previously [36]. As a result, 6 : 1 was considered the optimum molar ratio of the reactants for this reaction.

Then, the effect of different temperatures on LA conversion was studied. Similarly, the other three variables were fixed. As shown in figure 2, the LA conversion slightly increased when the temperature was increased from 55 to 70°C. The highest LA conversion of 97.11% was obtained at 70°C. However, an increase in temperature from 70 to 100°C reduced LA conversion. Then, the conversion slightly changes with the temperature from 100 to 115°C. This could be because the increasing temperature increased the rate of methanol evaporation, which ultimately influenced the esterification reaction [37]. The IL catalyst [Hnmp]HSO_4_ was dissolved in the reaction mixture at the test temperature; thus, it can effectively catalyse the reaction. The thermal stability of ILs mainly depends on the IL structure (i.e. cation/anion type) and cation modification (chain length, substituent number, C2 methylation and functionalization) [38]. The decomposition temperature could change from 200 to 400°C by varying the anion type, which indicates that anions play the most important role in determining thermal stability; [HSO_4_]-based ILs (with [BMIM] or [BPy] cation) are moderately stable (300°C ≤ *T*_onset_ < 350°C) [39]. In this study, the ILs used were [Hmim][HSO_4_] and [Hnmp][HSO_4_], the cations of which are [Hmim] and [Hnmp], respectively, and the anions are [HSO_4_]. Therefore, the ILs [Hmim][HSO_4_] and [Hnmp][HSO_4_] are stable at temperatures in this experiment. Furthermore, studies on pyrrolidinium-based ILs are limited and need further investigation. Therefore, to reduce the energy use of the process, 70°C was selected as the optimum reaction temperature.

Finally, the effect of different reaction times on LA conversion was studied. The effect of reaction time on LA conversion is shown in figure 2. The LA conversion increased noticeably up to 97.41% with increasing reaction time and remained stable thereafter. This could be because the methanolysis reaction approached equilibrium after 2 h, which explains the reason that the conversion of LA did not increase when the reaction time was prolonged further.

The LA conversion rate in this study is not significantly different from that in our previous study without removing water during esterification [23]. This may be because the esters produced during the reaction are not soluble in ILs and because ILs have strong water absorbability; i.e. they can absorb the water generated by the esterification reaction [40]. In addition, the reaction temperature was only 70°C in our present study. The steam cannot escape into the water separator, and the esterification reaction can remain only near the equilibrium conversion rate.

### 3.3. Response surface methodology experiments and study

#### 3.3.1. Regression equations and analysis of variance

Factorial experimental design has been extensively used for optimization because it reduces the number of cumbersome experiments, which in turn minimizes the consumption of the laboratory prepared catalyst. Furthermore, ANOVA methods are effective in the analysis of variables. The independent variables and their levels for the Box–Behnken design are given in table 1. To verify the models, 17 sets of experiment were required, and the obtained response values are shown in table 3. Table 3 shows that there was no observable difference between actual values and predicted values. Based on the data in table 3 and the quadratic equation model in equation (2.4), the relationship between the yield of biodiesel and the process variables was expressed by the following equation:

3.1 $$Y=95.43+3.27X_{1}+8.62X_{2}+4.32X_{3}-1.55X_{1}X_{2}-0.29X_{1}X_{3}-1.63X_{2}X_{3}-12.31X_{1}^{2}-7.16X_{2}^{2}-6.34X_{3}^{2},$$

where *X*_1_, *X*_2_ and *X*_3_ are the coded values of the test variables amount of catalyst, methanol/LA molar ratio and reaction time, respectively, whereas *Y* is the response of LA conversion. In the equation, the terms *X*_1_, *X*_2_ and *X*_3_ had a synergistic effect on the response value due to the positive sign in front of those terms, while other terms had a negative sign.

**Table 3.** Results of the response surface tests.

entry	variable and level			lauric acid conversion (%)	
	*X*_1_- amount of catalyst (wt %)	*X*_2_-molar ratio	*X*_3_-time (h)	experimental value	predicted value
1	1	0	1	84.37	84.08
2	−1	0	1	78.83	78.13
3	−1	−1	0	63.43	62.51
4	−1	1	0	81.54	82.87
5	−1	0	−1	68.61	68.90
6	0	−1	−1	66.74	67.36
7	0	0	0	96.91	95.43
8	0	−1	1	77.64	79.26
9	0	1	1	93.88	93.26
10	0	0	0	95.47	95.43
11	0	1	−1	89.48	87.86
12	0	0	0	94.20	95.43
13	0	0	0	95.38	95.43
14	1	0	−1	75.31	76.01
15	0	0	0	95.20	95.43
16	1	−1	0	73.48	72.15
17	1	1	0	85.38	86.29

As shown in table 4, statistical analysis based on ANOVA was used to estimate whether the quadratic model and model terms were significant or not by *p*-value. At the 1% level, the model *F*-value of 95.40, which was much greater than the tabular *F*-value (3.70), implied that the model was significant. The coefficient of determination (*R*^2^) of the model was 0.9919, which meant that the polynomial model was accurate and at least 99.19% of the variability in the data could be explained by the model. The value of the adjusted determination coefficient $\left(R_{\mathrm{adj}}^{2}=0.9815\right)$ was found to be high enough to support the high significance of the model. The adequate precision of 28.266, a measure of the signal-to-noise ratio, was much greater than 4, indicating adequate model discrimination. Furthermore, the relatively low value of the coefficient of variation (CV = 0.90%) demonstrated that the model had good precision and that the experiments carried out were reliable. Hence, this model was extremely significant, and the *p*-value of the lack of fit was 0.935, which was greater than 0.10, indicating that this model was reasonable. From these statistical tests, it was found that the model was adequate for predicting the LA conversion in the range of the variables studied.

**Table 4.** Variance analysis of the regression model.

source	sum of squares	d.f.	mean square	*F*-valve	*p*-valve	significant
model	1979.38	9	219.93	95.40	<0.0001	*
*X*_1_	85.35	1	85.35	37.02	0.0005	*
*X*_2_	594.95	1	594.95	258.06	<0.0001	*
*X*_3_	149.47	1	149.47	64.83	<0.0001	*
*X*_1_ *X*_2_	9.64	1	9.64	4.18	0.0801	
*X*_1_ *X*_3_	0.34	1	0.34	0.15	0.7138	
*X*_2_ *X*_3_	10.56	1	10.56	4.58	0.0696	
$X_{1}^{2}$	638.54	1	638.54	276.97	<0.0001	*
$X_{2}^{2}$	215.84	1	215.84	93.62	<0.0001	*
$X_{3}^{2}$	169.10	1	169.10	73.35	<0.0001	*
residual	16.14	7	2.31	—	—	
lack of fit	12.38	3	4.13	4.39	0.935	
pure error	3.76	4	0.94	—	—	
cor total	1995.52	16	—	—	—	

#### 3.3.2. Analysis of the response surface

**Figure 3.**
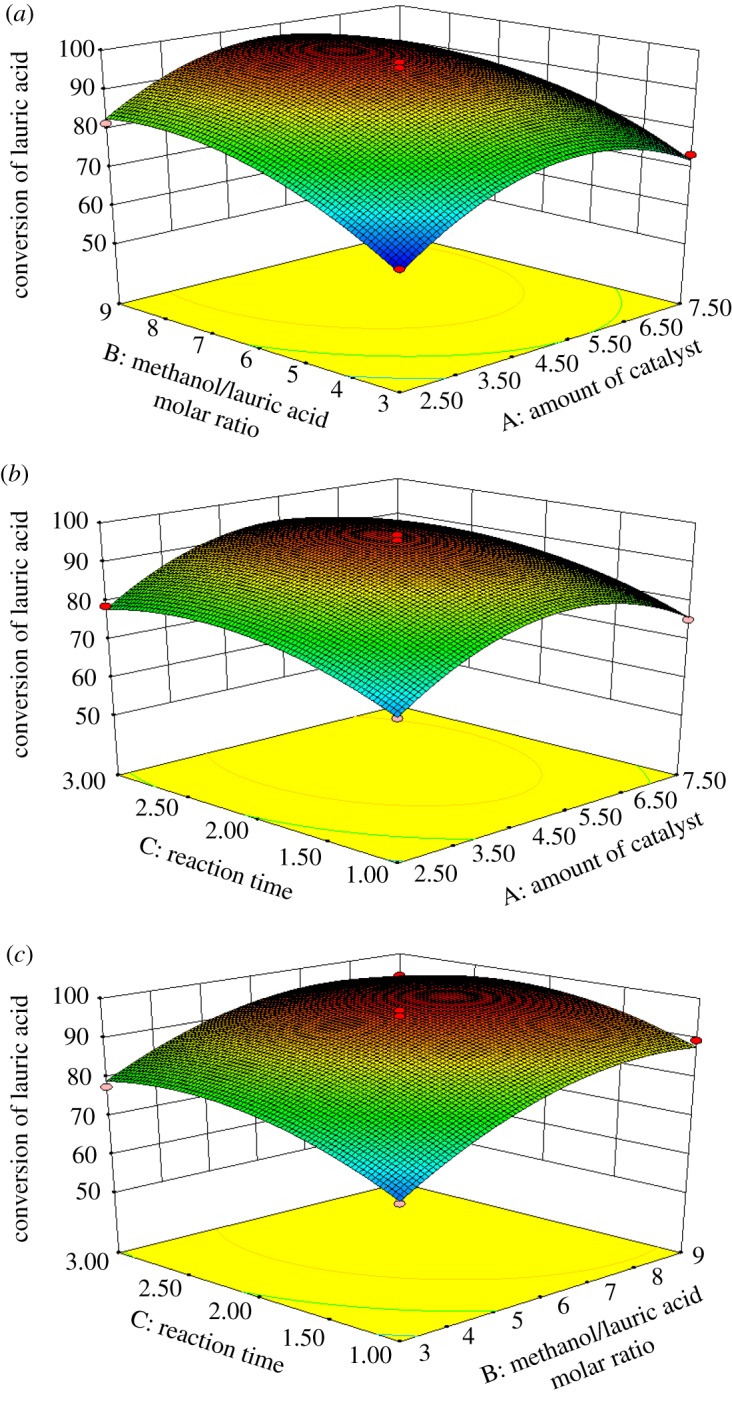
Response surface plots showing the predicted values of the LA conversion and the effect of (*a*) amount of catalyst and methanol/acid molar ratio, (*b*) amount of catalyst and reaction time and (*c*) methanol/acid molar ratio and reaction time, with the other variables held at a constant level.

The response surface corresponding to the regression equation is shown in figure 3. The three figures were all convex curves open at the bottom, illustrating the maximum response value and optimal conditions. The interactive effects of the [Hnmp]HSO_4_ dosage, molar ratio and reaction time on the LA conversion were analysed.

**Figure 4.**
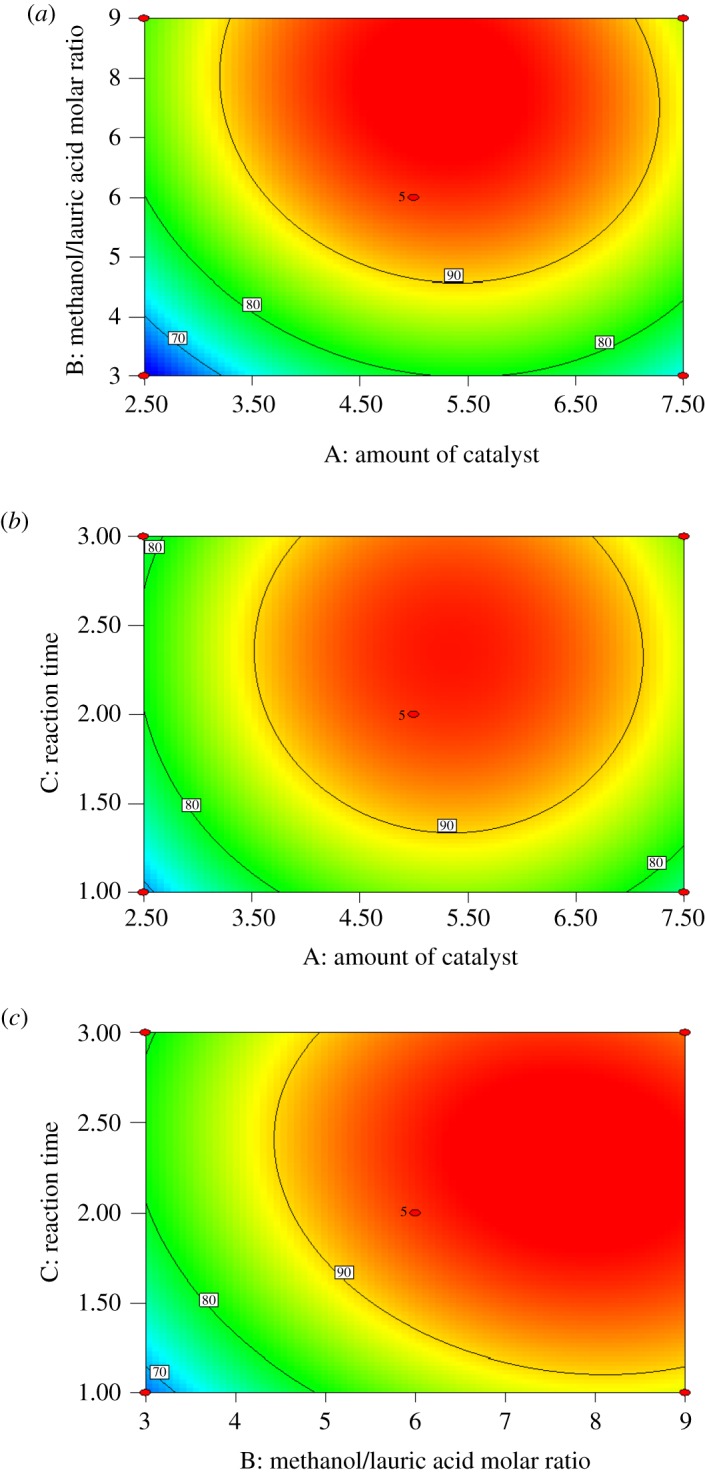
Contour plots showing the predicted values of the LA conversion, and the effect of (*a*) amount of catalyst and methanol/acid molar ratio, (*b*) amount of catalyst and reaction time and (*c*) methanol/acid molar ratio and reaction time, with the other variables held at a constant level.

The influence of each independent factor on the models was tested by analysing the variance at its level. According to table 4, the analysis of these parameters with the *p*-value indicated that the *X*_1_, *X*_2_, *X*_3_, $X_{1}^{2},X_{2}^{2}$ and $X_{3}^{2}$ terms had significant effects on the LA conversion. The three-dimensional response plots and contour plots of the amount of catalyst, methanol/acid molar ratio and reaction time are presented in figures 3 and 4, respectively. The three-dimensional surfaces are a graphical illustration of the regression equation. Each contour curve represented the combinations of two test variables while maintaining the other one at a level of zero.

The variations of LA conversion with the amount of catalyst and methanol/acid molar ratio are shown in figures 3*a* and 4*a*. The conversion varied with the amount of catalyst and methanol/acid molar ratio. The variation with different amounts of catalyst was obvious. The LA conversion first increased gradually to the peak value at a low amount of catalyst and then decreased with increasing methanol/acid molar ratio because the excess methanol, which diluted the concentration of [Hnmp]HSO_4_, resulted in decreased catalytic efficiency. In addition, the LA conversion increased quickly and then changed slowly with increasing [Hnmp]HSO_4_ dosage. It was evident that both the [Hnmp]HSO_4_ dosage and methanol/acid molar ratio exerted significant influence on the LA conversion [41]. The contour line with a symmetrical mound shape demonstrated that the combined effect of the amount of catalyst and methanol/acid molar ratio was not further significant.

The interactive effect of the amount of catalyst and reaction time is shown in figures 3*b* and 4*b*. With increasing time, more by-products were produced, and the conversion was low. After a period of time, the conversion changing slightly means that the effect of the amount of catalyst on the response was more obvious than the effect of time. The result was consistent with the values in table 4. The effect of the interaction of the two variables was not significant, with a symmetrical mound shape and a low *p*-value (0.7138) of the interaction term.

Figures 3*c* and 4*c* present the relationship between the methanol/acid molar ratio and reaction time. The trend was similar to the effect of the amount of catalyst and methanol/acid molar ratio. The LA conversion first increased and then decreased with time according to the methanol/acid molar ratio. The effect of the interaction of the two variables was also not significant with a symmetrical mound shape. The LA conversion was good at moderate reaction times and low amounts of catalyst.

Based on the comprehensive analysis of the response surface, we found that the interaction effects of [Hnmp]HSO_4_ dosage, methanol/acid molar ratio and reaction time were not significant parameters affecting the conversion of LA. According to the Box–Behnken design, the optimal conditions were as follows: [Hnmp]HSO_4_ dosage of 5.23%, methanol/LA molar ratio of 7.68 : 1 and reaction time of 2.27 h. The model predicted that the LA conversion could reach 98.58%.

#### 3.3.3. Verification of the regression model

According to the discussion above, it is possible to obtain a high LA conversion by searching for the optimum conditions. Hence, to test and verify these operations, a [Hnmp]HSO_4_ dosage of 5.23%, a methanol/LA molar ratio of 7.68 : 1, a reaction time of 2.27 h and a reaction temperature of 70°C were used. The LA conversion reached 98.35%, which confirmed that this model was reasonable.

### 3.4. Kinetics model

**Figure 5.**
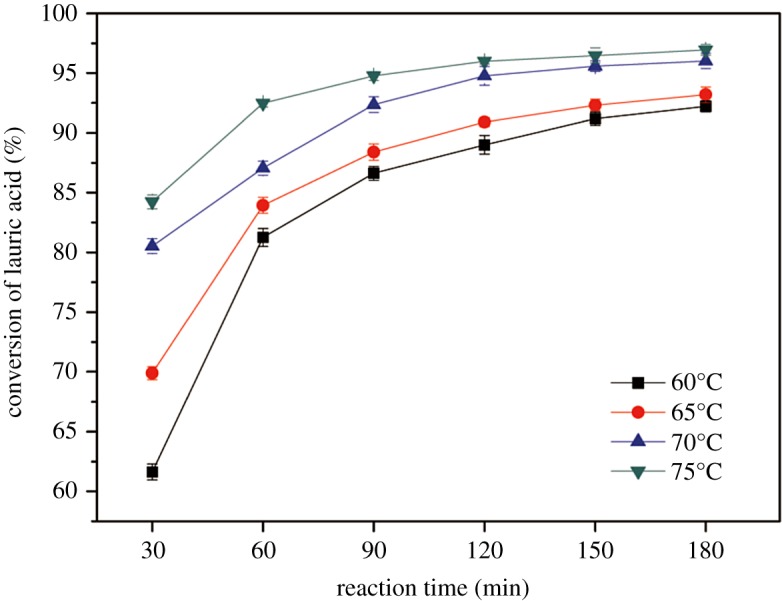
Conversion of LA at 60, 65, 70 and 75°C.

In this work, the esterification reactions were carried out under the optimized conditions, and the conversion of LA at different times and temperatures is shown in figure 5.

The experiments were performed at four different temperatures to investigate the conversion of LA with respect to reaction time. Figure 5 shows the comparison of LA conversion at 60, 65, 70 and 75°C. When using 5.23 wt% of [Hnmp]HSO_4_ in LA and a 7.68 : 1 methanol to LA molar ratio, the conversion of LA was found to increase with the reaction time. Clearly, the LA conversion rate was remarkably fast in the initial 120 min and then slowed up gradually from 120 min to 180 min. The conversions at 120 min for the reactions conducted at 60°C, 65°C, 70°C and 75°C were 89.00%, 90.91%, 94.77% and 95.98%, respectively. On the other hand, the LA conversion rate increased considerably with an increase in reaction temperature from 60°C to 75°C and reached the maximum conversion at 75°C. This could be because the methanolysis reaction approached equilibrium after 120 min, which explained why the conversion of LA did not increase when the reaction time was prolonged further. Based on the theory of Le Châtelier's principle, for endothermic reactions, the equilibrium shifts to the right as the temperature increases [42]. Therefore, the reaction temperature shows a positive effect on the conversion of LA.

**Figure 6.**
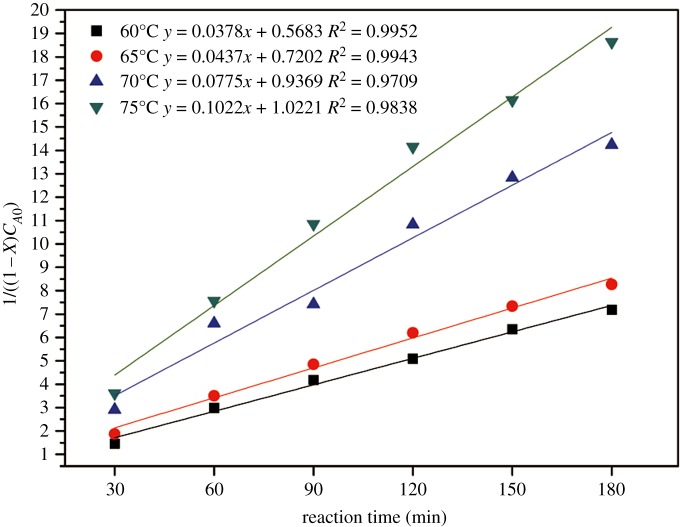
The linear relation of 1/((1 − *X*)*C_A_*_0_) with the reaction time at different temperatures.

Figure 6 indicates that the relations of 1/((1 − *X*)*C_A_*_0_) with time at different temperatures were straight lines, which implied that the kinetic equation of equation (2.8) for this esterification is correct. Figure 6 depicts the plots of 1/((1 − *X*)*C_A_*_0_) against *t*, where the slopes are equal to the reaction rate constants at four temperatures. The reaction rate constants are 0.0378, 0.0.0437, 0.0775 and 0.1022 g mol^−1^ min^−1^, corresponding to 60, 65, 70 and 75°C, respectively. The rate constants increase with the increase in temperature from 60°C to 75°C. This is because the reaction is endothermic and the forward reaction accelerates as the temperature increases.

**Figure 7.**
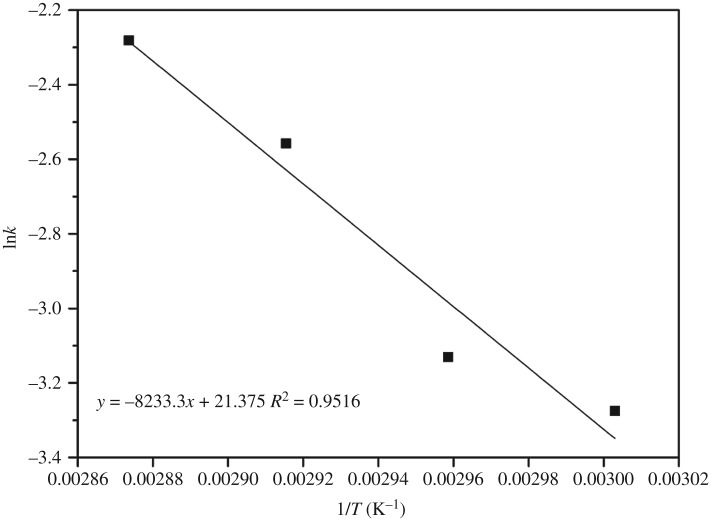
Arrhenius plot for estimation of the activation energy and frequency factor.

The influence of the temperature on the reaction rate was determined by fitting *k* to equation (2.10). Figure 7 shows the plot of ln*k* versus 1/*T*. The regression line was found to be linear with a high regression coefficient of 0.9516. From the slope and the intercept, the activation energy and the frequency factor are calculated to be 68.45 kJ mol^−1^ and 1.9189 × 10^9^ min^−1^, respectively. The relation between the equation and reaction rate constant and the temperature is described as follows:

3.2 $$-r_{A}=-\frac{\text{d}C_{A}}{\text{d}t}=1.9189\times 10^{9}{\text{e}}^{-6.845\times 10^{4}/RT}C_{A}^{2}.$$

The activation energy and frequency factor values of the esterification process from different experiments are shown in table 5. The results show that the value of the activation energy of [Hnmp]HSO_4_ is close to that in the case of ZnL_2_ [43] and is higher than that in the case of H_2_SO_4_ [42,44] and [BMIM][FeCl_4_] [45]. The frequency factor for this work is notably high (1.9189 × 10^9^ min^−1^), indicating that the reverse reaction can be neglected when a significant excess of alcohol (methanol or ethanol) is used [42]. This result indicates that the mass transfer in the reaction is easy to carry out due to the use of IL [Hnmp]HSO_4_. Compared to catalysts in previously reported work, the present catalyst exhibits higher activation energy depending on the reaction conditions and operates under mild conditions.

**Table 5.** Activation energy and frequency factor of esterification processes from different studies.

feedstock	catalyst	activation energy (kJ mol^−1^)	frequency factor (min^−1^)	references
lauric acid and ethanol	ZnL_2_	67.96	1.183 × 10^5^	[43]
oleic acid and methanol	H_2_SO_4_^a^	50.745	2.869 × 10^6^	[44]
oleic acid and methanol	H_2_SO_4_^b^	44.559	3.913 × 10^5^	[44]
oleic acid and methanol	[BMIM][FeCl_4_]	17.97	181.62	[45]
*Ceiba pentandra* seed oil and methanol	H_2_SO_4_^c^	53.717	3.98 × 10^9^	[42]
lauric acid and methanol	[Hnmp]HSO_4_	68.45	1.9189 × 10^9^	this work

^a^5% H_2_SO_4_ loading.

^b^10% H_2_SO_4_ loading.

^c^Microwave-assisted.

### 3.5. Recycling of [Hnmp]HSO_4_

**Figure 8.**
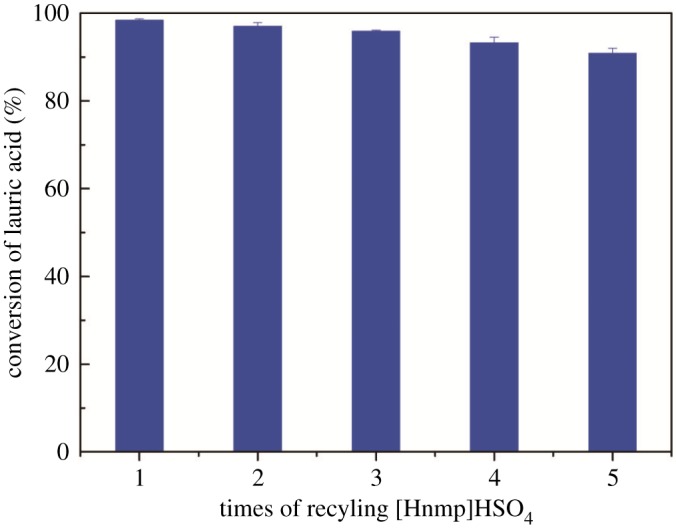
Effect of the number of cycles of the catalyst on the LA conversion.

To reduce the cost of the experiment, the possibility of recycling [Hnmp]HSO_4_ was investigated. The recycled IL was used in each cycle after the removal of water and unreacted methanol. After five recycles, the data shown in figure 8 indicate that there is little decrease in catalytic activity. The esterification rate of LA was still above 95%, which was in accordance with the literature [23]. Its performance in the esterification demonstrates the outstanding activity and excellent operational stability of the [Hnmp]HSO_4_ catalyst. However, as catalysts for the esterification of FFAs, other ILs, such as [NMP][CH_3_SO_3_] and [BMIM][HSO_4_], could be recycled eight times and five times, respectively [20,46].

## 4. Conclusion

This study indicated that IL [Hnmp]HSO_4_ is an effective catalyst for methyl laurate synthesis. The esterification procedure could be carried out at mild temperature, and the esters produced could be isolated conveniently. Response surface tests indicated that the optimal conditions were as follows: [Hnmp]HSO_4_ dosage of 5.23%, methanol/LA molar ratio of 7.68 : 1, reaction time of 2.27 h at 70°C; under these conditions, the LA conversion reached 98.35%. Moreover, a simple kinetic model was proposed, and the kinetic parameters were estimated by regression analysis. The activation energy and the frequency factor were determined to be 68.45 kJ mol^−1^ and 1.9198 × 10^9^ min^−1^, respectively. Notably, the catalytic activity of [Hnmp]HSO_4_ was still high after five cycles. Therefore, the process could become a viable alternative to the conventional process due to the uniqueness of the IL [Hnmp]HSO_4_ as a green catalyst, and could provide reference for the scale-up esterification of LA.

## Data accessibility

Data are available from the Dryad Digital Repository: http://dx.doi.org/10.5061/dryad.22mb72d [47].
